# Modeling Real-Life Urban Sensor Networks Based on Open Data

**DOI:** 10.3390/s22239264

**Published:** 2022-11-28

**Authors:** Bartosz Musznicki, Maciej Piechowiak, Piotr Zwierzykowski

**Affiliations:** 1 Faculty of Computing and Telecommunications, Poznań University of Technology, 60-965 Poznań, Poland; 2 Institute of Computer Science, Kazimierz Wielki University, Chodkiewicza 30, 85-064 Bydgoszcz, Poland

**Keywords:** urban sensor networks, open data, opportunistic routing, graph modeling

## Abstract

Epidemics and pandemics dramatically affect mobility trends around the world, which we have witnessed recently and expect more of in the future. A global energy crisis is looming ahead on the horizon and will redefine the transportation and energy usage patterns, in particular in large cities and metropolitan areas. As the trend continues to expand, the need to efficiently monitor and manage smart city infrastructure, public transportation, service vehicles, and commercial fleets has become of higher importance. This, in turn, requires new methods for dissemination, collection, and processing of data from massive number of already deployed sensing devices. In order to transmit these data efficiently, it is necessary to optimize the connection structure in wireless networks. Emerging open access to real data from different types of networked and sensing devices should be leveraged. It enables construction of models based on frequently updated real data rather than synthetic models or test environments. Hence, the main objective of this article is to introduce the concept of network modeling based on publicly available geographic location data of heterogeneous nodes and to promote the use of real-life diverse open data sources as the basis of novel research related to urban sensor networks. The feasibility of designed modeling architecture is discussed and proved with numerous examples of modeled spatial and spatiotemporal graphs, which are essential in opportunistic routing-related studies using the methods which rely on graph theory. This approach has not been considered before in similar studies and in the literature.

## 1. Introduction

Along with mobility restrictions, COVID-19 has drastically affected public transportation networks around the world. At its peak, ridership was reduced, revenue from ticket sales plummeted (in some cities, the decline in passenger numbers exceeded 90%) [1], and there were costs associated with disinfecting and implementing physical distance measures on public transport vehicles and infrastructure. The pandemic also influenced services and industrial production, including a halt in operations and a drop in turnover in the supply industry. Capacity management measures helped maintain the required capacity of public transport vehicles and stations. For instance, the increasing public transport frequency makes up for reduced capacity, reduces queues, waiting times, and overcrowding. At the same time, global health-promoting policies are promoting active lifestyles, as well as means of personal transportation. More and more autonomous vehicles, which are part of the infrastructure and generate large amounts of telemetry data, are appearing in public spaces. The development of fixed sensing infrastructure elements does not fall behind, growing in numbers and complexity of provided city-related data.

Typical wireless sensor networks (WSNs) consist of multiple nodes deployed over a certain area to perform a common sensing-related task [2]. The basic components of such networks are devices equipped with sensors that monitor the variability of physical phenomena and quantities, such as humidity, temperature, pressure, radiation, sound, motion, the degree of particulate air pollution, etc. Typical actual applications of sensors in urban networks include, but are not limited to, coordination of specialized vehicles (ambulances, emergency vehicles), public transport logistics (to optimize traffic and use of institutional resources), traffic management, monitoring of environmental parameters determining air and water quality, and monitoring of urban rental vehicles (electric cars, scooters, bicycles).

WSNs were and are focused on the optimization of wireless communication by implementing efficient algorithms and routing methods to save energy, among others. Such networks show the capacity to self-organize, resist single node damages, and apply radio transmission error correction and avoidance mechanisms. Every WSN node is equipped with measurement sensors, but also with its own power supply, wireless communication module, a microcontroller or microprocessor, memory, etc., i.e., such a node is a specialized computer and router that continuously processes measurements and routes data. Objects such as these may be combined to form an integrated system and are capable of cooperating to complete more complex and context-related tasks [3,4]. They are often combined with additional analytical tools and distributed resources provided by cloud computing [5]. Therefore, designing compact and energy-efficient network nodes is a challenge.

There is an ever-increasing potential for applications of devices based on various types of sensors, which are already seen as one of the elements of internet of things (IoT). The concept of IoT extends the idea of WSNs and creates an ecosystem that gathers measurement data and transmits them over different types of networks, and includes a range of elements such as cloud-based aggregation services, big data analysis, and network management tools. At the same time, more subcategories with clearly defined functions and purposes are emerging [6], includining vehicular sensor networks (VSNs), body area networks (BANs), home automation, smart factory, or smart city. However, they all have one thing in common—they measure and rely on wireless communication [7]. Thus, the boundary between a typical sensor network understood as a distributed measurement and communication system using often homogeneous resources with common radio technology and other studied types of wireless networks is blurred, especially in 5G networks [8]. Therefore, in a more general manner, all such heterogeneous measurement nodes can be called *connected* sensors [9,10,11].

Research on topology control of wireless sensor networks is focused on modeling and analyzing methods that can potentially be used to optimize the interconnection structure (e.g., to reduce power consumption). However, in practice, the ideas and concepts proposed by the research community are rarely used by network designers, and sensor systems that have already been implemented or are under development in urban environments rarely take advantage of the available research models developed and presented in the literature. Moreover, the widespread access to a variety of wireless technologies, which enable the empirical development and popularization of new solutions, especially in urban environments, also encourages the consolidation of this particular trend [12]. Analyzing the topology of such networks, designing new methods, and diagnosing problems should therefore be based on data obtained from real heterogeneous networks.

Sensor networks are often modeled using graphs in a similar way to modeling wired and wireless telecommunications and data communications networks. Most relevant WSN studies conducted hitherto have been mainly based on synthetic models and involved simulations [2] or the results obtained in experimental testbeds [13]. A number of other studies used historical data obtained from transportation operators. Fortunately, new data sources have started to arise in the last few years. Many of those are open (publicly available) and provide real-time open data on the location of public transportation vehicles and different elements of urban infrastructure as well as the readings of the measurements they perform. Usually, those application programming interfaces (APIs) are well documented and available to use free of charge, as discussed in Section 4.

The main objective of this paper is to promote the development and usage of publicly available real-life diversified data sources as the cornerstone of a novel urban WSN-related research approach. This approach is based on actual node data and location, as opposed to relying on synthetic models or limited homogeneous historical data, and paves the way for modeling of realistic space- and time-changing graphs that can be visualized, stored, and analyzed to design and optimize various aspects of the networks. This paper is, to the best knowledge of the authors, the first attempt to model and analyze dynamic heterogeneous urban sensor networks as graphs based on real node location data.

After the general introduction in this section, the second section presents what a sensor node and a sensor network currently mean in urban environments. In this section, types of urban sensor networks (Section 2.1) and characteristics of urban sensor nodes (Section 2.2) are presented. The third section of the article is focused on the presentation of key routing-related research problems in those networks. The fundamental issue of topology modeling and graph representation is discussed (Section 3.1), followed by the presentation of the even more essential matter of opportunistic routing (Section 3.2) and associated problems, i.e., data aggregation (Section 3.3) and data offloading (Section 3.4). The fourth section investigates the characteristics of urban node data sources and presents introduced network modeling architecture. In this section, crucial aspects of data availability (Section 4.1), data providers (Section 4.2), data formats (Section 4.3), data structure (Section 4.4), data scope (Section 4.5), and data update frequency and quality (Section 4.6) are analyzed. Then, data gathering, processing, and network modeling architecture are introduced (Section 4.7). They are used in the research proof of concept presented in the fifth section, where different feasibility proofs for using available open data for network modeling and routing design in urban environments are presented. Spatial graph modeling examples (Section 5.1) include the problems of modeling a static connectivity graph (Section 5.1.1) and a static minimum spanning forest (Section 5.1.2). Next, more complex spatiotemporal graphs are discussed (Section 5.2), beginning with modeling of a dynamic connectivity graph (Section 5.2.1), followed by space-time connectivity graph (Section 5.2.2), first-contact graph (Section 5.2.3), opportunistic localized class-based multicast tree (Section 5.2.4), and spatiotemporal shortest paths (Section 5.2.5). The article concludes with the summary and suggested directions for further research in this particular area.

## 2. Sensor Networks in Urban Environment

At present, one can observe an interesting evolution of sensor networks and the penetration of research areas in the study of these networks. The latest research areas do not provide findings that would solve the existing problems in homogeneous wireless networks and do not consider them separately for each type of network topology. The continuous development of radio technologies requires a holistic view of such a heterogeneous network of interconnected sensors, such as urban networks, where sensors operating in different wired and wireless technologies generate huge amounts of data. Moreover, different types of control and maintenance messages are propagated. In such networks, measurements and data access are fundamental.

### 2.1. Types of Urban SenNot Applicable.sor Networks

In the canonical wireless sensor network model, the objective and focus are on monitoring environmental parameters and efficient transmission of data collected in the monitored area (space) using low-emission, low-power wireless communication technology. The information is then relayed, through intermediary nodes, to an endpoint (designated as a controller or monitor) that processes it locally, as well as conveyed further through a portal (gateway) to various systems and networks, e.g., the Internet. Nodes can be fixed (stationary) or mobile and have a defined role and purpose. They tend to be envisaged to operate within a defined framework and, at least to some extent, a homogeneous and well-defined topology.

Wireless ad hoc networks (WANETs) are usually not bound to a strict framework or infrastructure. Therefore, they include numerous sub-types such as wireless mesh networks (WMNs), mobile ad hoc networks (MANETs), and vehicular ad Hoc networks (VANETs). The protocols used in MANETs are more complex due to mobility of nodes, and, thus, high network topology dynamics. At any time, network nodes can move spontaneously and in ways that are difficult to predict. Thus, the topology of an MANET can change rapidly and randomly at unpredictable times, which makes the design and implementation of routing methods a demanding task, hence the great popularity and a number of research works on MANETs and VANETs [14,15,16]. MANETs, as peer-to-peer multi-hop networks, assume the existence of mobile interconnected nodes which do not rely on any additional network infrastructure, such as base stations. As a result, there is no fixed infrastructure and the only limitation is the radio range of each network node.

VANETs are similar to MANETs in the sense that they do not require any infrastructure for data transmission. However, VANETs place more emphasis on responding quickly to changes in network topology, directly related to varying road traffic structure and density. They also support higher transmission speeds as compared to MANETs. Their optimization problems take into account the traffic patterns of mobile devices (vehicles) and accurate positioning (radar, nearby devices sensing, satellite positioning, etc.). In this sense, they are better suited to urban environments. They can also play an important role in safe driving, smart navigation, rescue, and entertainment applications. Therefore, VANET-based applications are widely used in urban environments. Due to the large amounts of data generated, the concepts of bandwidth-limiting, discarding redundant data, and prioritizing users during high-traffic scenarios were introduced [17].

The picture of the structural complexity of different types of urban wireless networks that have sensing capabilities may be completed by investigating various specialized and targeted applications. An example, presented by [18], is a wireless body area network (WBAN) that connects independent nodes (e.g., sensors and actuators) that are placed in clothing, on the body, or under the skin of a person. The network typically extends over the entire human body, and the nodes are connected via a close-range wireless communication channel. Depending on the implementation, the nodes are arranged in a star or multiwire topology [19]. Such a body area network (BAN) offers many promising new applications in the medical field: remote health monitoring, healthcare, multimedia, sports, and many others, all of which enable free movement of a BAN user. Moreover, the applications can be related to everyday and leisure activities when based on data gathered by smart watches and smart phones. Such networks are yet another source of the vast amount of wireless data filling up shared urban wireless transmission medium.

### 2.2. Characteristics of Urban Sensor Nodes

**Figure 1:**
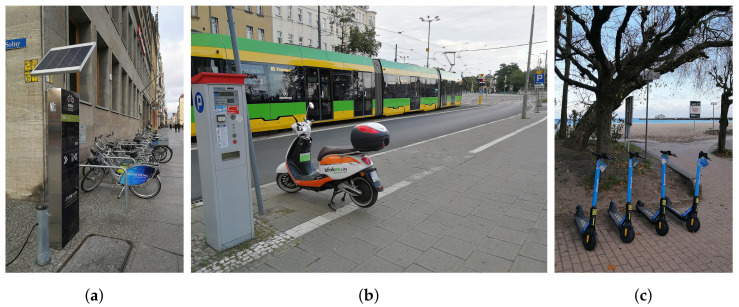
Examples of connected devices in cities in Poland. (**a**) Bike rental station in Wrocław on 10 December 2019; (**b**) parking meter, tram, and scooter in Poznań on 4 August 2019; (**c**) electric kick scooters in Sopot on 29 November 2019.

The possibilities for processing and generating data of various types by different types of nodes in heterogeneous urban environments are becoming essentially limitless. Most of those nodes perform, or could perform, some kind of sensing activity. Bike rental stations in Wrocław, parking meters, trams and scooters in Poznań, and electric kick scooters in Sopot are only a few examples of such connected devices already deployed in Polish cities, as presented in Figure 1. What can be pointed out, though, is the essential network and topological functions of a sensing node. In terms of routing, a single node can act as a source (originator), destination (sink), or relay (router). More complex relays are often termed (mobile) agents and are capable of performing advanced functions, such as data aggregation or buffering.

For instance, the paper [20] proposes a mechanism for node redundancy by introducing mobile agents that communicate opportunistically with a large field of sensors. The addition of mobile agents shifts computationally complex tasks from simple and power-constrained sensors to more advanced and efficient mobile agents. An increase in energy efficiency was achieved by adding an agent and modifying radio transmission at the physical layer. Not only dedicated sensor nodes can be mobile agents; so can other wireless devices with different energy and data processing properties carried or installed on vehicles (e.g., smart phones, sensor nodes on private vehicles and public transportation, laptops, and even sensor-equipped animals).

The mobility model should mimic the changes occurring in the actual network. A number of mobility models for ad hoc networks have been defined in the literature, along with analytical approaches to single node and group mobility. The paper [21] shows that in order to incorporate network dynamics into a mobility model, for example, the gradient descent method can be used in the optimization process (instead of the popular Newton’s law of motion known from classical dynamics). Synthetic networks with mobility models are used in the literature to test new routing algorithms and protocols for sensor networks [22].

The current state of research also takes into account the benefits of a heterogeneous architecture of wireless sensor networks, consisting of several mobile nodes of high performance and resources, accompanied by a large number of simple static nodes [23]. Mobile agents can act as mobile relays or mobile sinks. The performance of these two options and the trade-offs associated with them are being studied. Above all, the agent’s mobility for network discovery and efficient data collection from static nodes is being planned. Clustering schemes of hierarchical WSN architectures that use mobile relay nodes to achieve energy savings and extend network lifetime are also analyzed. Relay nodes are clustered again when failures are detected [24].

Even more challenges arise when researching solutions for distributed opportunistic wireless networks that are designed to be disruption- and delay-tolerant or resistant. Such networks, commonly termed delay-tolerant networks (DTNs), with (a number of) nodes having not only complex processing capabilities, but also buffering (caching) functionalities, are composed both of fixed (infrastructure) relays that perform *store-and-forward* functions, while mobile nodes (agents) perform *store-carry-and-forward* functions. Such mobile agents are frequently called data mobile ubiquitous LAN extensions (MULEs) [25]. In this way, network discontinuities may be bridged by delaying forward (relay) operation to a more suitable point in time, i.e., when new connectivity possibilities are provided due to the movement of the agent. In the case of city and traffic monitoring applications, this role can be played by vehicles (cars, buses, taxis, etc.) equipped with onboard network nodes and sensors. It is assumed that MULEs are capable of short-range wireless communication and can exchange data with a nearby sensing device or network gateway as they move past it. In this way, advanced mobile relays can receive data from sensors, buffer them, and send them to wired access points when they are in their vicinity. They can also operate in the opposite direction, i.e., disseminate data bundles, such as control messages from the source, to selected sensor nodes. The movement of some agents, such as drones, may be controlled, scheduled, or programmed to support their main functions, i.e., data routing. Other mobile relays, such as animals, humans, or vehicles, move regardless of their routing role and perform their network functions opportunistically. Movement patterns of some agents that follow preplanned routes and schedules can be predicted though, at least to some extent (e.g., for public buses and garbage trucks).

## 3. Routing Research Problems in Urban Networks

The complexity of data transmission issues in sensor networks is the source of many research problems. Those that relate to open (street) urban environment most are problems related to topology modeling, opportunistic routing, data aggregation, and data offloading, as discussed in the next subsections.

### 3.1. Topology Modeling and Graph Representation

The terms *graph* and *network* are often used interchangeably throughout this paper, but it is necessary to point out that a graph here is a mathematical structure used to model the topology of a communications network. A *graph* is defined as a pair G=(V,E), where *V* is the set of vertices (nodes) and *E* is the set of edges (links). Corresponding elements of a modeled *network* are devices and wireless connections between those devices. Each node and edge may be labeled with more than one parameter, such as identifier, occurrence time, or cost. The graph-based network modeling architecture introduced in this paper is presented in Section 4.7.

Although no methods for heterogeneous network modeling based on dynamic open data related to node locations existed so far, a number of more general, time-changing graph representations can be found in the literature. In short, they involve a time-ordered series of connected static graphs, a single graph with all node occurrences (instances) present as distinct vertices linked over time, or a single graph with node and edge attributes modeled as time series. To familiarize the reader with this domain, a brief overview of the general concepts and terms used is presented in the next paragraphs. They will be used as a starting point and naming reference, to keep present research description consistent with the graph theory domain.

Harary and Gupta (1997) suggested that a *dynamic graph* can be modeled as a sequence of static graphs [26]. Ferreira et al. (2003) use the name *evolving graph* for an analogical idea of a time-indexed sequence of sub-graphs as the formal abstraction for *dynamic networks*. Here, each sub-graph corresponds to network connectivity at a given time interval. The time domain is used in this model to restrict paths in the graph from moving over edges which were possible only in past sub-graphs [27]. Li et al. (2017) call the *dynamic network* a *temporal network*, i.e., an ordered sequence of separate networks which consist of the same set of nodes [28].

Merugu et al. (2004) construct a layered *space-time graph*, where each layer refers to a discrete time interval in the observation period of a network. Each layer has one copy of every node in the network with the consecutive copies being linked by directed temporal edges. Traversing a temporal edge corresponds to carrying a message by a node. Separate nodes are connected with spatial edges and traversing those denotes forwarding a message from one node to another [29]. Huang et al. (2011) used this concept to model and investigate *time-evolving networks* in the DTN context. Each graph of this sequence of static graphs is called a *snapshot*, and the space between consecutive layers of a resulting *space-time graph* is named a *time slot* [30].

George and Shekhar (2008), in a study on *spatiotemporal networks*, call a layered graph a *time-expanded graph*, as opposed to a *time-aggregated graph* [31], which is a directed equivalent of a *temporal network* postulated by Kempe et al. (2000). Here, a *temporal network* is an undirected graph with edges annotated with time labels that indicate in which time interval the communication between nodes took place [32]. For Holme and Saramäki (2012), an *interval graph* is the one whose edges are active over a set of intervals [33]. Correspondingly, a *temporal graph*, as defined by Wu et al. (2014), consists of vertices connected by edges labeled with starting and edge traversal times [34]. Flocchini et al. (2009) investigated the usage of *time-varying graphs* whose links are defined by periodic movements of mobile agents in the context of *dynamic networks*, whose topologies change frequently in time [35].

Not only the aforementioned original papers, but also the introductions, reviews, and discussions of different aspects of various temporal graph and network modeling methods are available [36,37,38,39].

### 3.2. Opportunistic Routing

Routing protocols designed for traditional multi-hop networks are designed for topologies that do not change that continually, frequently, and rapidly as the structure of heterogeneous and highly mobile sensor networks. Therefore, the process of designing efficient routing methods for WSNs is a particularly difficult task. Unicast, multicast, and broadcast algorithms and protocols need to be tailored to large-scale and frequently changing topologies of a varying node number and connectivity. Moreover, when designing methods for sensor networks, the requirement for energy conservation, especially when autonomously powered, as well as proper management of node resources, must be taken into account. As a result, the choice of the best data transmission path is more complicated than just a simple selection of one of neighboring successive nodes. Where a path is not always available, which cannot be known in advance, the network topology is expected to be partitioned and change frequently, with limited routing information available, while opportunistic routing may be implemented. The construction of a number of sample routing graphs is discussed in Section 5.2.4 and Section 5.2.5.

Opportunistic networks are an interesting step in the evolution of wireless networks. In opportunistic networks, source and destination might be able to communicate even if no time-continuous (uninterrupted) multi-hop path exists between them. Opportunistic routing benefits from the changes in the topology, usually related to node movement, and aims at bridging connectivity gaps by radio coverage extension or by message buffering.

One of the techniques currently used is to take advantage of the broadcasting nature of the wireless transmission medium. It can be assumed that omnidirectional radio transmission of one node can be overheard by multiple neighboring nodes simultaneously. Unlike popular routing mechanisms that select the next node before sending data, based on fixed parameters and network topology, opportunistic routing selects the next node or nodes dynamically once communication opportunities arise. In this way, forwarding of data may be then performed by the neighbor closest to the destination. Additionally, multipath and multiple-copy (*n-copy*) routing may be deployed to increase the probability of successful delivery. Opportunistic networks can even operate without, or based on scarce, routing information, implementing simple flooding-like methods [40] or more sophisticated ones, such as *beacon-less routing* [41], where no node presence messages (beacons) are periodically broadcast to make neighboring nodes aware of the existence (and often location) of other nodes. Opportunistic routing has been shown to achieve better performance than traditional routing under a variety of demanding conditions. One of the key tasks outlined in [42,43] may be the selection of forwarding nodes and the prioritization of nodes in this set.

The concept of opportunistic networks originated from the research on delay-tolerant networks, which led to the development of a DTN architecture. It typically consists of independent network partitions where there are only occasional opportunities for communication between them, sometimes known and scheduled in time, and sometimes completely random. The disconnected and dispersed networks form DTN regions, and the agent and gateway system are responsible for enabling connections between them. This model fits the characteristics of those urban sensing solutions which do not require fixed cell-like wireless network infrastructure or complete area-wide coverage. They may also be of use in emergency situations and conditions, such as natural disasters, grid power outage, or in a war zone. In such scenarios, single devices and separate islands of fixed (e.g., air quality meters) or mobile (e.g., humans with smartphones) nodes equipped with different sensors and radio interfaces are able to receive messages and transmit collected telemetry data and their location when in contact with a mobile agent or passing by a fixed gateway equipped with long-range network connection (e.g., a bus stop) [43]. Opportunistic routing can also support emergency services in everyday operations. The work of [44] analyzed Global Positioning System (GPS) tracks generated by fire service vehicles. The results reveal the characteristics of such networks formed by devices following this type of mobility with different radio communication ranges. Formed heterogeneous networks are scattered and fragmented, but there are delay-resistant routes connecting the areas. These results can be used in the design and implementation of solutions from the physical layer to the application layer.

Musznicki et al. [45] conducted the analysis related to the use of mobile and residential wireless local area network (WLAN) access nodes as opportunistic relays in the Wielkopolska region (Greater Poland) in June–July 2016. The study focused on a network of over 20 thousand fixed residential access points, 10 stationary commercial base stations, and 330 vehicular gateways. Mobile nodes equipped with cellular network connection were mounted in public buses and trams in Poznań and Konin. Residential gateways were implemented as add-on virtualized functionality to home wireless routers of an internet service provider. This *community WLAN* service enabled network access for the members of the community outside of their own apartments, when they were in the radio range of another router. Both types of access nodes were launched to provide opportunistic network access to authorized end nodes. To gather even more data in this closed commercial network, 10 stationary core base stations of significant coverage were selected. Together with the mobile relays, they were used as sensors measuring WLAN radio frequency noise floor and the strength of received signal transmitted by end nodes. The usage potential of residential nodes for the stationary WLAN network coverage extension was evaluated with the use of a mobile agent, i.e., a smartphone measuring various connection quality parameters while traversing residential areas in Poznań.

### 3.3. Data Aggregation

Smart city solutions aim at increasing sensing coverage, diversity, and quality of data obtained from various sensors to provide better services. The data are usually transmitted in a star-like topology, at least locally, to designated fixed nodes, i.e., base stations or gateways. The resources of such nodes can far exceed, in terms of computing power, available memory and storage, power supply, and connectivity, those of simpler devices. Therefore, they can act as transmission aggregators, local buffers, and relays to higher layers of network topology using dedicated connections (i.e., other transmission media and technologies or radio links of longer transmission range). However, covering entire metropolitan area with static sensors and providing continuous network access can be unfeasible or expensive. As a result, a few studies consider using a public transportation system as a mobile platform for sensor nodes with networked bus stops acting as data sinks [46]. Structure of this type can improve sensor network coverage and take advantage of opportunistic communication. This solution might be used in latency-constrained applications, and hence an algorithm was proposed to select which bus stops should act as sinks to minimize the maximum delay of message delivery. Experiments show that when using only 16% of bus stops as receivers, a nearly 10% increase in the maximum network delay can be achieved without significantly losing the spatial coverage.

Another example is the concept of the Internet of Bikes (IoB), i.e., a sensor network based on an urban bicycle system [47]. An IoB-DTN routing protocol based on data aggregation is proposed, which applies the DTN paradigm to Internet of Things applications. Data read by bicycles are transmitted in *store-and-forward* mode and aggregated by a set of dedicated receivers. The IoB-DTN protocol can be viewed as a simplified version of various *n-copy* DTN protocols, optimized for IoT devices (including some routing functions that are of no use in DTNs being removed).

Exemplary data aggregation related network is modeled in Section 5.1.2.

### 3.4. Data Offloading

Yet another research aspect is the problem of the huge amount of data generated by sensors in WSNs, especially in urban environments. Traffic generated by machine-type communication (MTC) devices reached 49 exabytes between 2016 and 2020 [48]. WSNs are typically said to include a large number of devices deployed randomly in a highly dynamic environment. The types of sensing capabilities, data gathering, and communications range of the nodes are typically fixed. When no DTN or opportunistic routing approaches are involved, high device density is used to maintain preferred level of network coverage to ensure the reliability of data collection. Oftentimes, the devices are event-based systems that may attempt to report occurring events or perform measurements and transmit data at the same time. Therefore, attention was placed on optimizing the amount of data transfer in such wireless networks. In [49], the authors proposed a low-latency, low-power medium access control (MAC) protocol for hierarchical wireless networks. The protocol involves the transmission of data from end nodes to the sink node via a cluster head. However, this approach applies to large, hierarchical but homogeneous networks, while, as outlined earlier, in real-life urban networks, the boundaries between node types and transmission technologies are blurred.

Bonola et al. [50] conducted data dissemination and gathering scenarios analysis based on position traces of about 320 taxi cabs in the center of Rome, Italy, in a six-month period in 2013–2014. The area of 8 km by 8 km was characterized by congested narrow roads of high traffic and low speeds. It was divided into a 200 by 200 cell grid, where each cell covered the area of 40 m by 40 m. A *store-carry-and-forward* approach was investigated, in which taxis incidentally passing stationary nodes, such as trash bins and street lights, exchanged data with them and performed the roles of data MULEs. The results of the study indicated that with 120 vehicles on average, 80% coverage can be achieved in less than 24 h. A one-month portion of gathered taxi mobility traces is publicly available at *CRAWDAD* repository [51].

Similarly, Dias et al. [52] investigated the feasibility of a delay-tolerant vehicle network in the city of Rio de Janeiro, Brazil, using public transportation system data. The performance of such a network was evaluated by analyzing a large dataset of high mobility data—12,456 buses and 5833 taxi cabs recorded over a 24 h period based on their GPS positions. The presented results indicate the validity and feasibility of the use of the public transportation system as a delay-tolerant data network that provides significant coverage of the city. In the study, a clustering algorithm was used to group nearby vehicles into cells. Then, those clusters were modeled as nodes of a weighted directed graph with edges representing vehicle travels between clusters, and a number of metrics were analyzed. It is worth mentioning that the data were collected by the authors in October 2014 from the source available through open data portal *Data.Rio* [53]. The data were then shared by the authors and can be downloaded from *CRAWDAD* repository [54]. They contain recorded date and time, identifier, line, latitude, longitude, and speed of each bus.

A method for modeling a data offloading related graph is presented in Section 5.2.3.

## 4. Sources of Urban Nodes Location Data and Network Modeling Architecture

The variety of node types and routing research problems in sensor networks operating in urban environments is followed by the growing diversity of real-life and real-time data sources. In spite of each source having its own unique features, a number of more general characteristics can be distinguished, as listed and described in the following sections. Based on these, a generalized urban sensor network modeling architecture can be introduced, as presented in Section 4.7.

### 4.1. Data Availability

The most basic division of digital data source types one can currently think of is related to whether the data are available online or offline, as well as whether the data are provided in real time or as an archived dataset.

Online and offline repositories of historical data (archives) may be of great value, especially when they provide rich research data. The challenge with such repositories is that they are currently few in number and provide selected datasets, such as the ones in the *CRAWDAD* repository mentioned in Section 3.4. Some enable access only to partial, or not necessarily up-to-date data [55], while others require formal efforts and agreements or do not provide any access to external researchers at all.

The present study suggests that there are no heterogeneous frequently updated high-quality data archives available to urban sensor network researchers. Hence, as long as no such repositories are in existence, the use of publicly available online data, referred to as *open data*, shall be considered. Such sources offer much easier access, usually to real-time or quasi-real-time data, and are more promising in terms of novel research areas and approaches. Moreover, similarly to closed offline archives, there exist numerous systems that could provide real-time online access to valuable *closed data* if they are approved by a responsible authority.

Real-time sources gradually grow in numbers and the data are usually provided based on REpresentational State Transfer (REST) [56] APIs. To obtain the data, first an HyperText Transfer Protocol (HTTP) GET request needs to be sent to a particular resource address, i.e., a Uniform Resource Locator (URL), often called an endpoint. Then, as a result, an HTTP response containing the requested dataset is returned by the server. Sources of this type are of interest in the next sections. Less frequently, the data are provided as more static files that need to be downloaded and unpacked. Gathered data can be used in a real-time application, as well as stored for further usage in a solution-specific custom-made repository (e.g., the one used in the present research and described in Section 4.7).

### 4.2. Data Provider

The data are, or can be, provided by a number of entities which maintain and manage measurements gathering and processing systems. They include global communities and commercial enterprises, as well as regional and national authorities, public services providers, etc.

**Figure 2:**
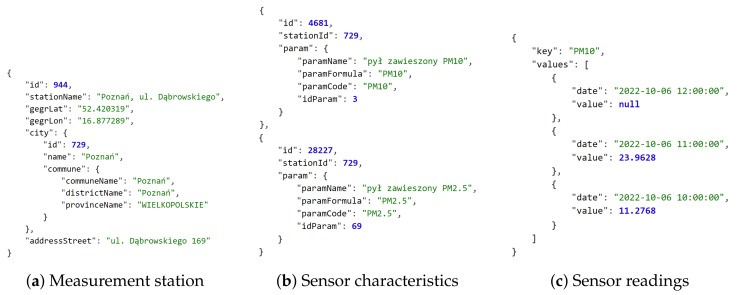
Examples of the data provided by GIOŚ *Air Quality* portal on 6 October 2022 [61].

A good example of the above is the *Packet Broker Mapper* API which enables open access to the data related to the global IoT ecosystem of *The Things Network*, providing the locations of LoRaWAN gateways, number of their antennas, online status, etc. [57]. Then, there are the open data related to stationary air quality measurement stations in Poland provided by commercial providers *Airly* [58] and *Syngeos* [59], as well as the *Chief Inspectorate of Environmental Protection (GIOŚ)*. The *Air Quality* portal operated by this national authority provides access to both archived [60] and real-time data API [61]. Example responses of this portal are presented in Figure 2.

There is also an increasing number of regional authorities that operate their own open data websites and portals related to public services [53,62,63]. Very often, the Comprehensive Knowledge Archive Network (CKAN) open-source software [64] is used at the core of those portals [55,65,66]. This management system allows data sources to be grouped, described, and presented in a user-friendly way. Each dataset can be made available in a number of data formats and enriched with metadata and access methods examples. CKAN operators can use the *DataStore* extension to automate data update and retrieval processes [67]. Once a resource is updated, its preview page can be automatically refreshed with the *Data Explorer* extension, to be ready to be presented to the end users. Moreover, *DataStore API* enables users to search, filter, and fetch the data without having to download the whole dataset. Hence, a *PostgreSQL* query can be used, for example, to limit the number of returned records, parameters, or even to convert their data types.

It needs to be pointed out that open data are currently a relatively limited source of information, as compared to closed data systems. Such systems, that belong to smartphone manufacturers, navigation software providers, etc., operate based on much larger, diversified, and constantly changing volumes of data related to the location and operation of each device in the network. Mobile nodes, in general, tend to generate larger amounts of measurement data than the fixed ones. A good example of heavily monitored solutions is the segment of vehicles used in the innovative solutions for *shared mobility* (also called car-sharing, bike-sharing, etc.), which is a new, distinct, and evolving category of urban mobility [68]. It includes various types of vehicles for individual and commercial use (e.g., cars, bicycles, scooters) [69,70,71,72]. This transportation strategy allows users to gain short-term paid access, on an *as-needed* basis, to various types of vehicles widely dispersed throughout the city, such as the bikes and scooters presented in Figure 1. Once the trip is completed, the vehicle becomes available for subsequent users. Gathered measurements are primarily accessible to and used by the operators of such networks with no open data access provided. Although, based on special agreements, access may be granted to interested third parties, e.g., to the developers of mobile applications, such as *take&drive* [73], which aggregate and present the data form multiple sources, i.e., related to numerous urban means of transportation. Moreover, other types of car fleets, trucks, and service vehicles (e.g., ambulances, police cars, and garbage trucks), as well as taxis and delivery vehicles, are monitored as well. Depending on local and national regulations, access to closed data related to public infrastructure and service vehicles, in particular to the archived location records, might be possible, upon request, based on the right of a citizen to access public information [74].

### 4.3. Data Format

**Figure 3:**
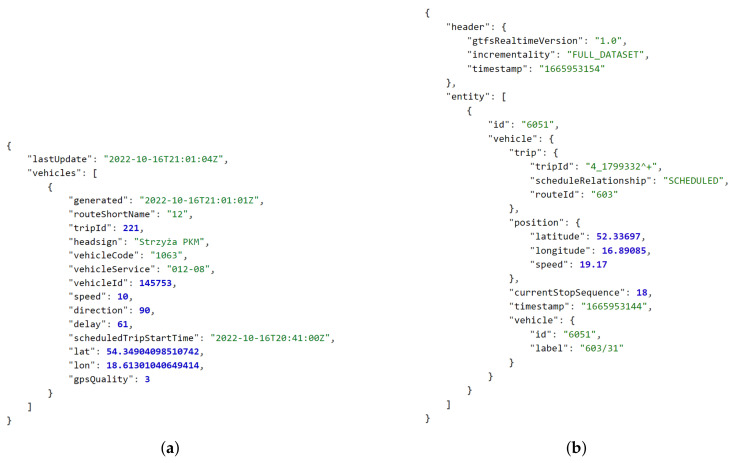
Samples of public transport vehicles data in two Polish cities on 16 October 2022. (**a**) Tram in Gdańsk [78]. (**b**) Bus in Poznań [79].

The most common data export formats used by open sources are textual. Currently, the leading format is the JavaScript Object Notation (JSON), which is a data-interchange format (syntax) based on the object literals of JavaScript programming language [75], see Figure 2 and Figure 3. A far less frequent format is the Comma-Separated Values (CSV) [76], which can sometimes be enabled as an alternative to JSON access to CKAN-based data sources or to provide metadata related to a data source.

Far less frequent sources of interest, binary data exchange formats, are also being implemented. The one which is actively developed and increasingly frequently used is the *Protocol Buffers* mechanism [77]. This method for serialization and deserialization of structured data minimizes the size of the message being transmitted (or a *.pb* file), while preserving its full content. The original message is encoded and decoded according to the message type definition stored in a fixed template defined for a given protocol (a *.proto* file). In this way, in contrast to a JSON file, the structure of a binary message is minimized and contains mostly a numbered series of values which correspond to specific parameters defined in the template. Therefore, the *.proto* file needs to be used at the receiving side to decode the message, i.e., to determine field names and types.

### 4.4. Data Structure

It can hardly be said today that all sources follow the same well-established data structure. However, there are specifications to which source developers are increasingly turning to in order to standardize the matter. An example of JSON-based solution-specific convention is *GeoJSON* format, which was designed to represent geographic objects together with related attributes [80]. Another one, the *GTFS Realtime*, an extension to General Transit Feed Specification (GTFS), specifies the structure in which public transport operators shall provide real-time data related to their services. The specification includes the information on vehicle positions (location and congestion levels), trip updates (delays, cancellations, and route changes), and service alerts (unplanned travel or infrastructure events) [81]. The data are published in *Protocol Buffers*-based format.

Clearly visible, though, is the fact that numerous analyzed sources do not follow any common data structure. The hierarchy of the elements of a response differ, and usually, the meanings, naming conventions, and data types or accuracy of the elements vary. It can be seen in both CSV and JSON-based formats.

First, compare the structures of the public transport vehicle related data in Poland for Gdańsk (received as a JSON-based structure) [78] and Poznań (received as *Protocol Buffers*-based *GTFS Realtime* format and decoded to JSON) [79] presented in Figure 3. Gdańsk-related data are also available in the *GTFS Realtime* format, if required [82].

Then, please locate the position within the structure and the format of the timestamps:

- Figure 2c—*date* key related to *2022-10-06 12:00:00* reduced local time formatted string.
- Figure 3a—*generated* key related to *2022-10-16T21:01:01Z* extended complete UTC (*Coordinated Universal Time*) ISO 8601 formatted string [83].
- Figure 3b—*timestamp* key related to *1665953144* string in POSIX (Unix) time format [84].
- Figure 5b—no timestamp.

It also shall be noted that the latitude and longitude are not only given as different elements of the structure, but are sometimes of an unusual data type (i.e., a string):

- Figure 2a—*gegrLat* key related to *52.420319* string.
- Figure 3a—*lat* key related to *54.34904098510742* floating-point number.
- Figure 5a—*latitude* key related to *54.409971066405* floating-point number.
- Figure 5b—second element of *coordinates* array, i.e., the *52.412023* floating-point number.

Furthermore, as illustrated in Figure 5b, the language of the elements of the structure may be mixed. For example, the keys *type* and *street* are in English, whereas the payment methods *bilon* (coin) and *karta* (card) are in Polish, with Boolean values given as strings, i.e., *TAK* (YES) and *NIE* (NO).

**Figure 4:**
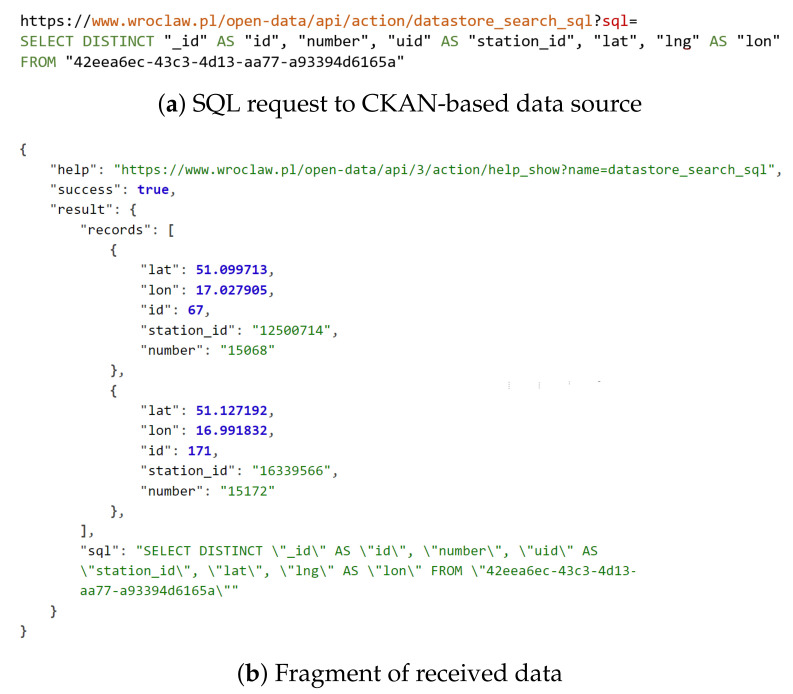
Server-side filtered and formatted Wrocław City Bike [85] on 16 October 2022.

Within the context of the presented differences, it is necessary to use a conversion software while gathering and processing data from a number of heterogeneous sources to standardize the data before further usage. To ease this task, the features of some CKAN-based sources may be taken advantage of to tune the structure and scope of the responses to the needs of the user using SQL-based queries. Figure 4 presents an example of such a request and response related to the data source [85] for rental stations of *Wrocław City Bike* [86]. The real-time information is filtered and shaped as the data consumer sees fit for the purpose. In this particular case, only unique (distinct) records were requested and received, the number of parameters (i.e., the scope of the data) was limited, and three original field names were altered by the server to reduce transmission overhead and computational efforts on the receiving side.

### 4.5. Data Scope

The scope of available data related to the elements of urban devices depends on both the decisions of the operator of the network and the nature and features of the device itself. Although different kinds of data are provided, one is of key importance in this study, i.e., the geographic location of stationary and mobile elements. The fundamental coordinates, latitude and longitude, are expressed in accordance with World Geodetic System ’84 (WGS 84) notation [87]. Other location-related parameters, such as altitude or street address, may be additionally present. Typically, some other application-specific features, such as the identifier, name, type, status, or capabilities of a node, are also parameterized.

**Figure 5:**
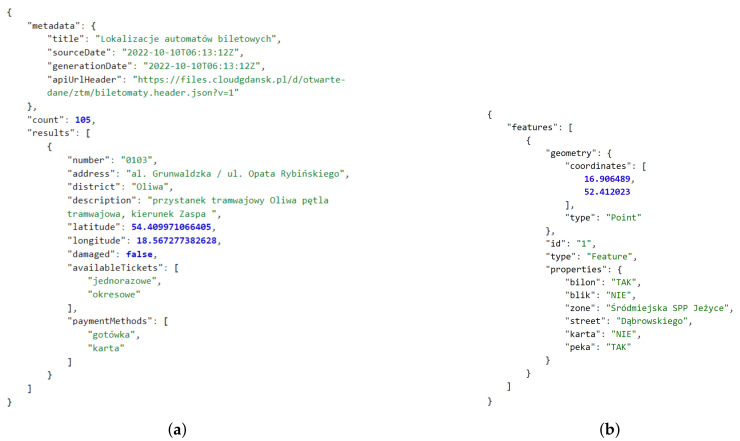
Ticket machine and parking meter data on 23 October 2022. (**a**) Ticket machine in Gdańsk [91]. (**b**) Parking meter in Poznań [92].

In case of public transportation, additional information may include current speed of the vehicle, side number, and route or trip identifiers, as visible in Figure 3. Furthermore, correlated information may be available to determine the vehicle type (e.g., low-floor), equipment (e.g., ramp, air conditioning, voice announcement system, ticket machine, and USB chargers), and whether space to carry bicycles is provided [79,88]. The timetables are commonly available as well [89,90]. Ticket machine and parking meter data can cover, for instance, district or zone name and supported payment methods, as in Figure 5. The air-quality-related data usually extend over the number of measurement types, such as particulate matter (floating dust) readings PM10 and PM2.5, presented in Figure 2.

What is crucial for real-time applications and data analysis is that each received dataset should be unambiguously timestamped—preferably at the source. Frequently, each record of a dataset is also timestamped—when the source distinguishes the moment of each obtained reading.

### 4.6. Data Update Frequency and Quality

Online urban data sources belong to one of the three categories in terms of update frequency—frequent (i.e., almost real time), less frequent, or archival. Some are well documented in this matter, such as the source of frequently updated locations of public transport vehicles in Warsaw, Poland, which is updated each 10 s [93]. The source related to the intelligent transportation system (ITS) in Gdańsk captures the data, while the resulting delay is approximately 20 s for each vehicle independently. If a vehicle loses the connection to the data-gathering server, its position is not updated and is discarded after 5 min [78]. Some other sources provide no commentary on the update frequency, and this needs to be concluded from the analysis of the changes of timestamps (time markers) included in the responses. It tends to be no more than 5–20 s. To see these kinds of data and network dynamics in action, i.e., changing and visualized over a city map, *BusLive* [94], a mobile application, can be used. It presents (almost) real-time locations of buses and trams in a number of Polish metropolitan areas.

Similarly to the information related to mobile nodes, stationary sensing node parameters are also frequently changing, as the measurements progress in time. Some of those sources impose though free-access frequency limitations. In the case of *Airly* [58] and *Syngeos* [59] air quality measurements networks, those are set to the maximum of 100 requests per day.

Information on fixed infrastructure elements, such as bus stops, parking meters, and ticket machines, is less frequently updated. One can usually expect these data to be updated at least once every 24 h [95]. It seems that few archival data sources group related data by year [60] or by day [90].

Due to the open data nature of the sources of interest, there are usually no service level agreements (SLAs) that would guarantee the quality and continuity of the data. This results in some sources producing, from time to time, corrupted or outdated data. Moreover, because of technical problems, some reading series may be missing when the service is not (fully) operational. In spite of data providers aiming at sharing data of required quality and continuity, each researcher needs to take into consideration the fact that quite often there is no quality guarantee and, as a result, countermeasures need to be put in place to cope with broken or missing data.

### 4.7. Data Gathering, Processing, and Network Modeling Architecture

**Figure 6:**
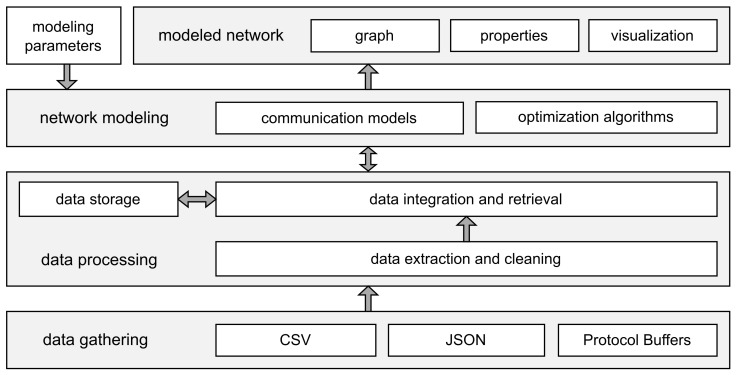
Open-data-based architecture for urban network modeling.

Due to the lack of ready-to-use heterogeneous historical node data, as well as no real-life data-based urban sensor network modeling architectures existing, the architecture presented in Figure 6 was proposed to address the research problem defined in Section 3.1. The data are obtained, processed, and a network is modeled in the following steps:

1. Data gathering:
  (a) Query each data source;
2. Data processing:
  (a) Extract and clean received data;
  (b) Preprocess, integrate, and store the data to local archive (data storage);
3. Network modeling:
  (a) Retrieve the data of interest from local archive;
  (b) Model network topology and connectivity as a graph based on given modeling parameters and node attributes (e.g., position and type);
  (c) Solve network optimization problem (e.g., find a tree);
  (d) Calculate the properties (attributes) of the resulting network (graph);
  (e) Save the graph in the archive for further use;
  (f) Visualize the network (with or without background city map).

Numerous sources of open data on the geographic location of different types of urban nodes were overviewed and analyzed as described in the preceding sections. As a result, Poznań, Poland metropolitan area related sources provided by *Airly* (air quality meters [58]), *Smart City Poznań* (public transport stops, parking meters, and ticket machines [62]),and *Public Transport Authority in Poznań* (real-time vehicle-related information [79]) were selected to be the input for the exemplary network modeling presented in Section 5.

To implement this approach, *Linux*, *PostgreSQL*, and *Python*-based data-gathering software was developed to automate the process of continuous data gathering from multiple open data sources, as well as extraction, cleaning, integration, and storage in a local archive. Then, a graph representation and analysis environment was built on top of *NetworkX*—a network analysis library which provides basic data structures for graphs and implements numerous standard operations and algorithms [96]. Finally, static and dynamic (time-changing) graph modeling methods were implemented and a visualization based on the map data from *OpenStreetMap* [97] was introduced. The performance of such software depends on both the computing capabilities of the local platform and on the response times and transmission delays of open data sources. Should the need arise to, for example, assess the robustness of different available computing platforms, a simple execution-time-based comparison may be used that compares the time needed to construct a given graph or a series of graphs from locally stored data.

## 5. Network Modeling Proof of Concept

The question to answer in the present feasibility study is whether diverse publicly available data sources and datasets can be successfully used for modeling and solving routing research problems in heterogeneous urban sensor networks. The modeled graphs and usage examples presented in the next subsections neither exhaust the scope of the research field nor provide universal and final solutions to the current routing-related research problems defined in Section 3. Their aim is to prove that the modeling architecture introduced in Section 4.7 can be successfully used to study real-life networks to improve and develop new data transmission methods.

### 5.1. Spatial Graph Modeling

A typical routing-related research study is based on monitoring and analysis of actual network traffic or a simulation in synthetic or semi-synthetic networks [98,99], as well as on the analysis of static or infrequently changing graphs [100]. The goal of the first part of the modeling proof is to show that a static spatial graph (a nontemporal graph) depicting a wireless network of sensing capabilities can be modeled and analyzed in the center of Poznań, a city of approximately 700 thousand inhabitants [101], during afternoon rush hours on Wednesday, 27 November 2019, at 3:15 p.m.

#### 5.1.1. Static Connectivity Graph

**Figure 7:**
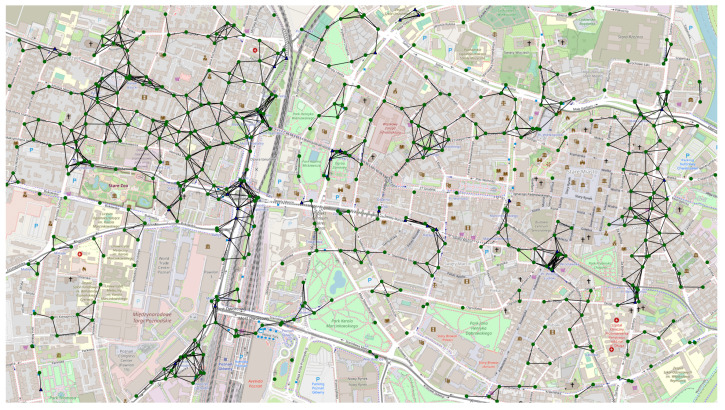
Wireless connectivity graph of a static sensor network modeled in the city center of Poznań on 27 November 2019.

A single static undirected radio connectivity graph, presented in Figure 7, was constructed by selecting gathered timestamped node location data that belonged to a single time interval and by determining assumed wireless links between those nodes (physical devices). This structure can be considered as the most basic graph representation of a momentary network topology, as described in Section 3.1. It consists of the green circle fixed (stationary) nodes (i.e., air quality meters, parking meters, public transportation stops, and ticket machines) and the blue triangle mobile nodes (i.e., buses and trams). The node coordinates (i.e., latitude and longitude) are defined following the WGS 84 notation. A link exists between two nodes if the geographical distance between them does not exceed the radio range. This distance is calculated using the haversine formula, which determines the great-circle distance between two points on a sphere [102]. The following modeling assumptions were made:

- Time interval: 6 s;
- Area dimensions: 3 km by 1.7 km;
- Area boundaries:
  - Minimum latitude: 52.400;
  - Maximum latitude: 52.415;
  - Minimum longitude: 16.898;
  - Maximum longitude: 16.942;
- Radio range: 100 m;
- Radio coverage: omnidirectional.

A network modeled in this way, i.e., a network *snapshot*, captures the state of the assumed physical wireless network structure in a given period, i.e., the interval of duration meaningful for the analysis, and can be the basis for various studies related to routing research, in particular, in solving the open problems summarized in Section 3. The degree of a node is determined by the number of other nodes within the radio range of that node. The network is denser, i.e., the connected components (sub-graphs) consist of more nodes and edges, in areas with a higher density of the infrastructure elements (ticket machines, parking meters, vehicles, etc.) and more busy street routes. It is clearly visible that the shape and structure are largely related to the layout of the streets and the distribution of the supporting fixed infrastructure. The resulting spatial graph is characterized by the following metrics:

- Nodes: 501;
  - Fixed nodes: 465;
  - Mobile nodes: 36;
- Average node degree: 4.02;
- Edges: 1008;
- Total spatial edge cost: 67,135 m;
- Connected components: 59.

#### 5.1.2. Static Minimum Spanning Forest

**Figure 8:**
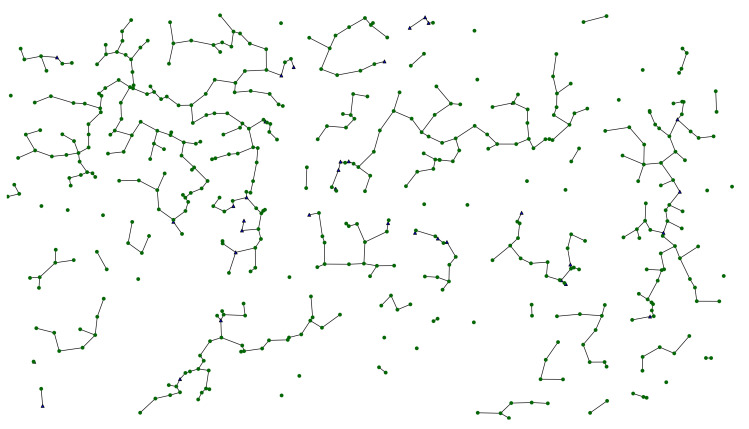
Minimum spanning forest of modeled static sensor network graph in the city center of Poznań on 27 November 2019.

In the next step, the minimum spanning forest (i.e., the set of minimum spanning trees determined for each connected component) of the static undirected graph in Figure 7 was determined using Kruskal’s algorithm [103], with the edge weight being the geographical distance between the nodes expressed in meters. This is presented in Figure 8, this time without the background city map to place more emphasis on the graph itself and its components. This forest can be described with the following basic parameters:

- Nodes: 501;
  - Fixed nodes: 465;
  - Mobile nodes: 36;
- Average node degree: 1.76;
- Edges: 442;
- Total spatial edge cost: 23,645 m;
- Connected components: 59.

The proposed spatial modeling approach may be of use in numerous research fields. For instance, it might be related to the determination of the optimal number and placement of stationary data aggregation gateways (sinks) in a data dissemination and collection network, as discussed in Section 3.3. In this way, a tree connecting all disconnected components might be constructed to model and mimic the hierarchical network topology, with such special-purpose nodes located in the centers of each network cluster (connected component) or between them.

### 5.2. Spatiotemporal Graph Modeling

To research realistic highly dynamic networks, especially in mobile urban environments, with a number of nodes and connections varying in time, time-changing spatial graphs can be studied. Although modeling of such spatiotemporal graphs poses numerous challenges, it can be achieved on the basis of accurate geographical location and telemetry data that can be available more and more often. To prove it, the graphs discussed in the next sections were constructed.

They can be used, for instance, in the studies on opportunistic mobile DTN data routing between network clusters or dispersed nodes to enable the delivery with minimum cost or delay. Moreover, in particular, when more mobile nodes data related to smartphones, cars, bikes, etc., are available, the research may involve the analysis of street traffic trends and determination of most busy (hot-spot) areas in the city at given time of day, as well as general-purpose smart city infrastructure planning.

#### 5.2.1. Dynamic Connectivity Graph

**Figure 9:**
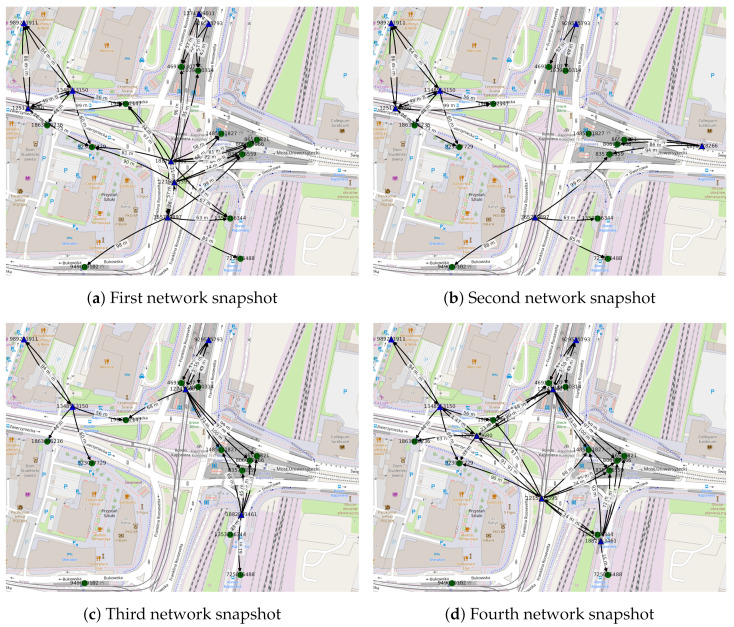
Opportunistic spatiotemporal sensor network evolving graph modeled in the vicinity of Kaponiera Roundabout in Poznań on 27 November 2019.

The first four *snapshots* of a *dynamic network* modeled as an *evolving graph*, i.e., a sequence of static connectivity graphs, is presented in Figure 9. Each connectivity graph depicts potential radio connections between the networked devices in the area of the Kaponiera Roundabout in the city center of Poznań. The whole directed graph spans 120 s on 27 November 2019, starting at 3:00 p.m. Similar to the static connectivity graph presented in Section 5.1.1, it includes stationary (i.e., air quality monitoring stations, parking meters, public transport stops, and ticket machines) and vehicular nodes (i.e., buses and trams). Each node was a single device with its precise satellite-positioning based location known. Not only geographic coordinates were provided by data sources, but also various additional parameters related to the nodes, such as the speed of each bus and tram, the names of the stops, payment methods supported by parking meters, or air quality readings. They were not needed, though, in this network dynamics and routing modeling proof of concept but could be used, for example, in prediction-based studies or environmental and social trends analysis. The modeling parameters were the following:

- Number of intervals (slots): 20;
- Time interval: 6 s;
- Area dimensions: 357 m by 272 m;
- Area boundaries:
  - Minimum latitude: 52.406511;
  - Maximum latitude: 52.408955;
  - Minimum longitude: 16.909878;
  - Maximum longitude: 16.915140;
- Radio coverage: omnidirectional;
- Radio range: 100 m;
- Relay nodes:
  - Mobile nodes: buses and trams;
  - Fixed nodes: air quality meters and parking meters;
- Destination nodes: public transport stops and ticket machines.

The green circles in Figure 9 are destination nodes and the blue triangles are the relays. The mobile nodes are marked with the black symbol border. The node labels (e.g., *323097729*) are unique identifiers of physical devices. The edge labels, such as 68 m, indicate the geographical distance between devices.

To model the network, first, the gathered data samples related to the instances of the nodes present in the area of interest in the analyzed period were discretized (i.e., grouped), according to their timestamps, into consecutive time intervals (slots) of chosen duration. This duration is the resolution of the modeling process and should be set to be at least as short (as high) as the update interval of the most frequently updated data source. To mimic the envisaged DTN application, the physical nodes were categorized into two sets—relay and destination nodes—based on their class, i.e., the device type and assumed network capabilities. Relays may originate connections with other devices, i.e., be the starting nodes for directed edges, while destination nodes can only be at the receiving end of a directed link. Then, similarly to the spatial graph modeling approach presented in Section 5.1.1, the radio connectivity graph was constructed for each of the time slots, based on the location, assumed radio range, and role of each node. The edges of such graphs shall be called spatial edges, being the representation of network connections and directed single message transmissions, modeled to be feasible in a given smallest considered time period (slot). The network modeled in this way can be analyzed and used to solve different topology and routing problems defined in Section 3, as presented in the next subsections.

#### 5.2.2. Space-Time Connectivity Graph

In this section, the *evolving graph* from Section 5.2.1 is transformed into a *space-time graph* (or a *time-expanded graph*). Every physical node which belonged to a slot is represented in this graph as two temporal sub-instances (nodes) connected with a directed edge. The first instance was related to the start of the slot and the second to the end of this slot. Next, each slot-end instance of the given physical node occurrence was connected with a more recent slot-start instance of this or a physical neighboring node in a following but not necessarily consecutive spatial graph (snapshot) with a directed temporal edge pointing towards the newer instance. Every vertex (physical node instance) was labeled with its slot number. Duration of a temporal edge was stored as temporal distance (cost) of an edge, while geographical distance was recorded as spatial distance (cost).

#### 5.2.3. First-Contact Graph

**Figure 10:**
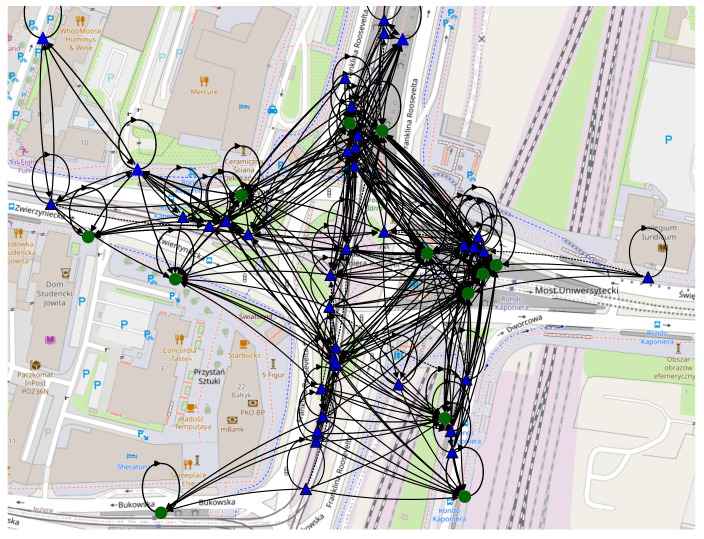
Space-time connectivity graph.

The produced layered graph depicted in Figure 10 consists of multiple temporal instances of physical nodes, each being a distinct graph node linked with a directed edge to the next temporal instance of it and to respective instances of other physical nodes. Such a graph preserves all information about spatial and temporal relationships between nodes—as opposed to a simple composition (addition) of nodes and edges of all spatial graphs, which would likely lead to misrepresentation caused by the existence of edges which would not be possible in the network if the direction (flow) of time was obeyed. Each node was visualized in the location of its first appearance in the graph. Visible self-loops mean that there was more than one node (instance) present in a given location. The key characteristics of the presented space-time connectivity graph are the following:

- Physical nodes: 26;
  - Fixed nodes: 15;
  - Mobile nodes: 11;
- Node instances: 714;
- Average node degree: 3.78;
- Edges: 1348;
- Total spatial edge cost: 41,580 meters;
- Time span: 20 slots.

**Figure 11:**
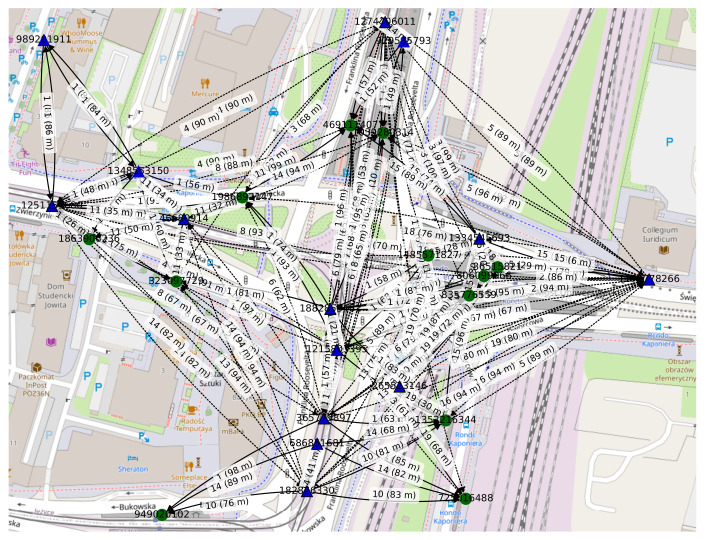
First-contact graph.

The *space-time connectivity graph* shown in Figure 10 can be transformed into a *first-contact graph*, presented in Figure 11. Such a graph can be viewed as a form of a *time-aggregated graph*. The procedure starts by removing all spatial edges between two physical nodes that are not the first-contact edges, i.e., that are not the earliest occurrence of an edge between those nodes. Next, each spatial edge is additionally labeled with its slot number. Then, all temporal edges connecting the instances of the same physical node are removed, as long as they point toward the instances that are later in time than the last (newest) instance connected to another physical node. Finally, all temporal instances of each physical node are composed (merged) into a single spatiotemporal node and their edges are connected to other respective composed spatiotemporal nodes.

The edge labels presented in Figure 11 depict the slot number in which the space edge existed between two nodes and geographical distance between them. The connections that take place in a given time slot, i.e., the spatial edges, are depicted as solid lines. Time-delayed spatial edges are indicated by dotted lines (i.e., a number of slots had to pass before the contact between two physical nodes was possible). For instance, the dotted edge labeled *5 (89 m)* means that a time-delayed connection existed in slot number 5 and the distance between devices was 89 m at this time. These are the parameters of the graph:

- Physical nodes: 26;
  - Fixed nodes: 15;
  - Mobile nodes: 11;
- Average node degree: 10.92;
- Edges: 142;
- Total spatial edge cost: 9949 m;
- Time span: 20 slots.

Because some of the time-order related information might be lost in the process, a *first-contact graph* will be of no use for searching the shortest spatiotemporal paths and solving related problems. It can be used, though, in the overall analysis of the contacts of selected nodes. With this approach, different graph and node parameters can be computed and investigated, such as the first-contact node degrees computed to select the nodes with the largest number of first contacts in the network. Hence, a graph of this type can be used in the design of data offloading mechanisms described in Section 3.4.

#### 5.2.4. Opportunistic Localized Class-Based Multicast Tree

The literature presents a number of algorithms for building multicast trees and methods for assessing their quality mainly for static networks [104,105]. The method for building multicast trees in opportunistic routing environments characterized in Section 3.2 can serve as another usage example. The objective of this process is to select spatial and temporal edges which allow a data bundle (or a message) to be transmitted (routed) within a given time period of interest, from the source node to all other nodes which belong to a particular class—here, a selected physical node type. Each bundle will be propagated along its own tree, and therefore the process can be called Single-Bundle Class-based Multicasting (SBCM). Each node is aware only of its local neighborhood, i.e., of other nodes within its radio range. This approach may be used, for example, to distribute software updates and controls or emergency messages to selected class of devices distributed throughout the urban area.

To model this type of *time-aggregated graph*, a time series of consecutive spatial graphs, i.e., *evolving graph* constructed in Section 5.2.1, is used as the input for a distributed opportunistic multicasting algorithm to construct a new graph—the opportunistic time-aggregated multicast tree. First, one of the relay-class nodes originates the bundle and becomes the root of the tree, as well as the first actual relay that stores this bundle and forwards it opportunistically. Then, in each following time slot, every relay which stores the bundle, i.e., every node that became a member of the tree, attempts to forward it to next relays or destination nodes, and hence new relay nodes are connected to the tree. To make routing decisions, each relay node uses its own local knowledge about past transmissions and its neighborhood, being aware of all other nodes in its radio range. Thus, every directed link between two nodes, either spatial or temporal edge, corresponds to a bundle transmission. It is assumed that such a transmission can successfully be completed within a single time slot and relay nodes operate with bundle buffers of unlimited capacity (infinite bundle queues). If a node already belongs to the tree, i.e., received the bundle, it will not be considered to be a recipient of future transmissions of this bundle, and therefore will be connected to the tree only once to store and forward or process the bundle, being a relay or a destination, respectively.

**Figure 12:**
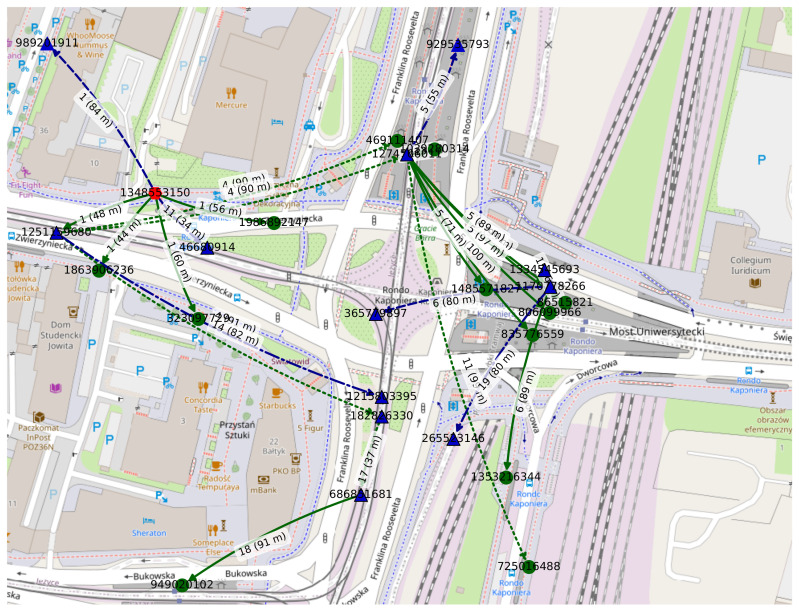
Opportunistic localized class-based multicast tree.

As shown in Figure 12, this algorithm successfully constructed a multicast tree, and the bundle could reach every destination node in this opportunistic routing scenario related to Section 3.2. Due to the opportunistic nature of the algorithm, there is a number of stub relay nodes that are connected to the tree but do not belong to the core part of it, i.e., do not lead to any destination nodes. In the visualization, the orange hexagon node labeled *1348553150* is the source, the green circles are multicast receivers (terminals), and the blue triangles are the relays. The mobile nodes are marked with a black border. The green edges are the edges that compose the actual (core) multicast tree, i.e., are the building blocks of the paths leading to multicast receivers. The blue edges are connections to the relays that do not lead to the terminals. The graph metrics are as follows:

- Physical nodes: 25;
  - Fixed nodes: 15;
  - Mobile nodes: 10;
- Multicast tree nodes: 25;
  - Core relays: 5;
  - Stub relays: 7;
  - Destinations: 12;
- Average node degree: 1.92;
- Edges: 24;
- Total spatial edge cost: 1685 m;
- Time span: 19 slots.

To avoid bundle buffer overflows or transmission medium oversaturation in this simplistic example, one could consider the implementation of the mechanisms such as time-to-live (TTL) of a bundle (expiration time), last-in, first-out (LIFO) limited capacity bundle queues, the selection of a smaller number of relays in denser networks, etc., or the use of more advanced protocols.

#### 5.2.5. Spatiotemporal Shortest Paths

Yet another usage area may be related to searching for globally optimal opportunistic shortest paths, based on full spatial and temporal knowledge on the structure of the graph, and, hence, network topology. Such knowledge can either be available *a posteriori*, i.e., after the observation period, or beforehand, to some extent, when prediction models that are sufficiently precise can be used. This is quite the opposite to the SBCM case, where only space- and time-limited localized topology-related knowledge is available to the routing algorithm.

What is crucial is that the presented method allows a *space-time graph* to be analyzed using well-known methods and tools designed for directed static graphs, sometimes only with minor modifications, such as one of the well-known shortest-paths-finding algorithms [106]. One can, for example, in an opportunistic routing case related to Section 3.2, look for the geographically shortest paths and the time shortest (fastest) paths [34], as well as the energy shortest paths between two nodes, provided such spatiotemporal paths exist [107]. Another aim to be achieved can be to construct a multicast tree that consists of the shortest spatial distance directed paths between the root (source) and the leaves (designated destinations).

**Figure 13:**
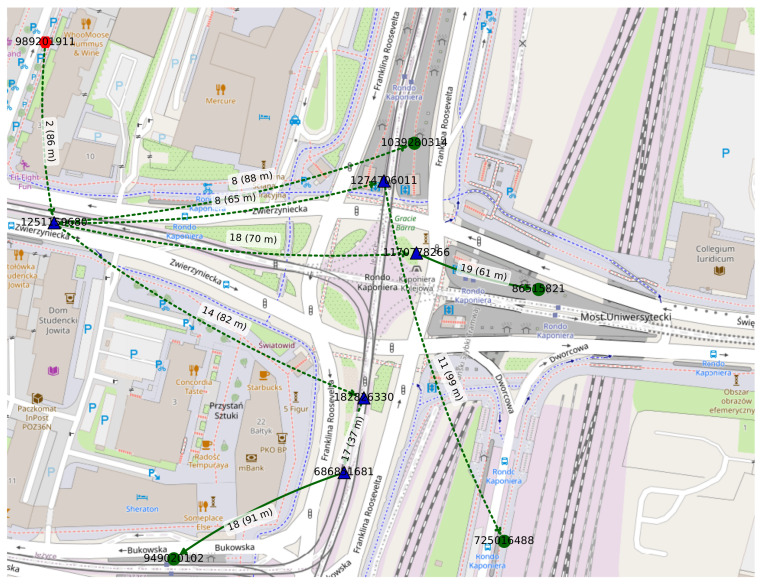
Opportunistic spatiotemporal shortest paths to selected nodes.

An example *time-aggregated tree* constructed in the *evolving graph* modeled in Section 5.2.1 using Dijkstra’s algorithm is presented in Figure 13. The source is depicted as the orange hexagon node labeled *989201911*, the five relay nodes are the dark blue triangles, and the four destination nodes are the green circles. The parameters of the tree are as follows:

- Physical nodes: 10;
  - Fixed nodes: 5;
  - Mobile nodes: 5;
- Multicast tree nodes: 10;
  - Core relays: 5;
  - Destinations: 4;
- Average node degree: 1.8;
- Edges: 9;
- Total spatial edge cost: 679 m;
- Time span: 20 slots.

## 6. Conclusions and Future Work

The deployment of connected sensors in urban environments and the widespread availability of data they provide opens up new areas of research and creates novel study opportunities. As demonstrated in this article, owing to publicly available online data sources, it has become possible to model real-life dynamic urban sensor networks. The introduced network modeling approach based on exact, time-varying location and number of heterogeneous sensor nodes has not previously been considered and presented in the literature. Captured topologies can be represented as accurate spatiotemporal graphs. In this way, the key research problems in the area may be studied, i.e., opportunistic routing, data aggregation, and data offloading, as proved by the presentation and discussion of numerous graphs modeled in the feasibility study. Therefore, the main goal of this paper was achieved. Moreover, the relationship and disparity between the theoretical network concepts and evolving urban structures is presented, followed by a brief characterization of nodes with sensing capabilities in actual urban networks. Furthermore, open data sources are thoroughly discussed and described, with samples of data they provide.

The results of presented preliminary routing-related research indicate the complex nature of network dynamics in urban sensor networks, and hence the introduced network modeling architecture, and static (spatial) and dynamic (spatiotemporal) graph models enable network researchers to perform various types of routing-related studies using graph theory methods. As presented, those may include the development of concepts for opportunistic routing in delay-tolerant networks based on actual geographic location of nodes. More generally, they can be used in the emerging real-life data-based research or play the key role in the incorporation of new ideas into well-studied routing concepts in MANETs, VANETs, WSNs, etc. Further research should first of all address the development of efficient and easy-to-implement algorithms aimed at optimal deployment of fixed nodes, the usage of large numbers of already available mobile devices, or design and optimization of routing algorithms, as well as data aggregation and radio spectrum and power-preserving schemes. All of them will aid applications such as low-powered everyday and distributed delay-tolerant measurements data collection or dissemination of messages during emergencies, pandemics, power outages, and natural disasters.

In future work, the authors intend to explore further the use of open data to study opportunistic routing and design of effective and efficient DTN topologies. Another significant research input will be a more detailed description, explanation, and best practices for node data gathering and construction of the presented spatiotemporal graphs. Study of the impact of the use of more advanced radio propagation and communication models might also be of interest.

## Data Availability

Not applicable.
